# An Integrated Engineering Approach to Creating Health‐Modulating Postbiotics

**DOI:** 10.1002/mnfr.70326

**Published:** 2025-11-17

**Authors:** Michael Leonidas Chikindas, Diana E. Roopchand, Santosh Kumar Tiwari, Liubov S. Sichel, Mukund V. Karwe, Nitin Nitin, Vitor Luis Fagundes, Igor V. Popov, John R. Tagg, Xuanxuan Lu, Svetoslav Dimitrov Todorov

**Affiliations:** ^1^ Health Promoting Naturals Laboratory School of Environmental and Biological Sciences Rutgers State University New Brunswick New Jersey USA; ^2^ Department of General Hygiene I.M. Sechenov First Moscow State Medical University Moscow Russia; ^3^ Department of Food Science, NJ Institute of Food, Nutrition, and Health Rutgers State University New Brunswick New Jersey USA; ^4^ Department of Genetics Maharshi Dayanand University Rohtak Haryana India; ^5^ Stellar Biotics, LLC Rockleigh New Jersey USA; ^6^ Department of Food Science and Technology University of California Davis California USA; ^7^ Programa De Pós‐Graduação em Ciências Veterinárias, Departamento de Medicina Veterinária Universidade Federal do Paraná Curitiba Paraná Brazil; ^8^ ProBacLab, Laboratório de Microbiologia de Alimentos, Departamento de Alimentos e Nutrição Experimental, Food Research Center, Faculdade de Ciências Farmacêuticas Universidade De São Paulo São Paulo São Paulo Brazil; ^9^ Center For Agrobiotechnology Don State Technical University Rostov‐on‐Don Russia; ^10^ BLIS Technologies South Dunedin Dunedin New Zealand; ^11^ Department of Food Science and Nutrition, Yip Kit Chuen Building The Hong Kong Polytechnic University Hung Hom Kowloon Hong Kong; ^12^ CISAS‐ Center for Research and Development in Agrifood Systems and Sustainability Instituto Politécnico de Viana do Castelo Viana do Castelo Portugal

**Keywords:** health benefits, inactivation, postbiotics, probiotics

## Abstract

Postbiotics have emerged as a promising alternative to live probiotics, offering comparable health benefits while overcoming challenges related to safety, stability, and shelf life. This review provides a comprehensive overview of the current state of postbiotic research, beginning with updated definitions and the rationale for transitioning from live microbial formulations to inanimate postbiotics. We examine the diverse mechanisms by which postbiotics modulate host physiology, including enhancement of epithelial barrier function, immunomodulation, systemic metabolic regulation, neuroactive effects, anti‐inflammatory activities, and anticancer properties. Detailed discussions highlight how bioactive components—such as bacteriocins, exopolysaccharides (EPS), short‐chain fatty acids (SCFA), and specific proteins (e.g., Amuc_1100 and P9 from *Akkermansia muciniphila*)—mediate these effects through complex cellular signaling pathways and host‐microbe interactions. Furthermore, we review the antimicrobial potential of postbiotic formulations, emphasizing their role in controlling pathogenic and spoilage microorganisms. Various methods for microbial inactivation are discussed, ranging from conventional thermal techniques (e.g., pasteurization and ohmic heating) to non‐thermal approaches (e.g., ultrasonication, ionizing radiation, and ultraviolet light), as well as innovative hybrid methods that combine chemical, physical, and enzymatic treatments. These strategies not only ensure the complete inactivation of live microorganisms but also preserve the integrity and bioactivity of postbiotic compounds.

Comparative analyses of live probiotics versus postbiotics reveal that inactivated formulations can deliver similar or even enhanced health benefits, with superior safety profiles and improved quality control. The review concludes by addressing current challenges in standardizing postbiotic definitions and production processes and by outlining future research directions necessary to unlock their full potential in clinical, nutritional, and biotechnological applications.

## Graphical abstract

Postbiotics have emerged as a promising alternative to live probiotics, offering comparable health benefits while overcoming challenges related to safety, stability, and shelf life. The review addresses current challenges in standardizing postbiotic definitions and production processes, and by outlining future research directions necessary to unlock their full potential.

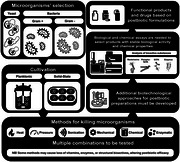

## 1 Introduction

Probiotics are defined as “live microorganisms that, when administered in adequate amounts, confer a health benefit on the host” [1]. While numerous studies have documented the beneficial effects of probiotics on humans and other animals [2], emerging evidence also highlights some potential adverse impacts. Serious complications—including cardiovascular infections, septicemia, and other health issues—have been reported primarily among immunocompromised individuals, young children, or the elderly with weakened immune systems [3, 4, 5, 6]. Moreover, certain strains may harbor antibiotic‐resistance genes, posing risks to animal health and the human food chain [7]. The unintentional introduction of insufficiently characterized strains into agricultural settings further raises concerns about ecological balance and biodiversity.

In addition to these risks, some adverse effects can be attributed to the bioactive substances produced by probiotics. Others may arise when these “friendly” microorganisms transition to an opportunistic or even pathogenic role under conditions of weakened host regulation—a shift that can also reduce overall microbiota diversity despite some evidence of microbiota support under specific conditions [8, 9, 10]. These observations underscore the need for rigorous strain selection, continuous monitoring, and deeper mechanistic studies to ensure probiotic safety.

**FIGURE 1:**
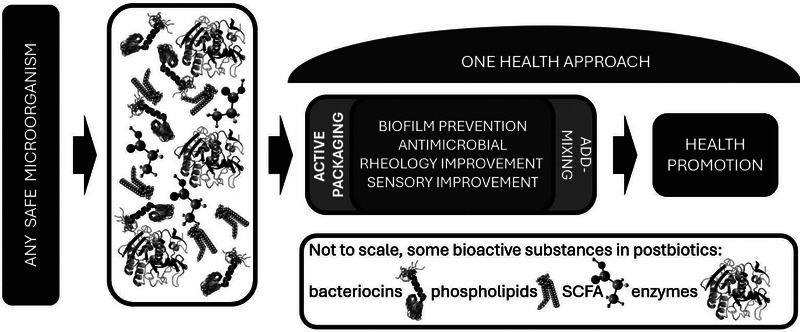
From selecting a safe microorganism, identifying beneficial properties and developing health promoting applications under the auspices of the One Health: Integrated Approach concept to defining the landscape in which the beneficial properties of microorganisms can be applied.

To address these challenges, researchers have increasingly explored postbiotics as an alternative approach. Postbiotics are defined as “preparations of inanimate microorganisms and/or their components that confer a health benefit on the host” [11] (Figure 1). Unlike live probiotics, postbiotics consist of inactivated cells, their metabolites, or cellular components—thus sidestepping issues related to viability during food processing and storage. It is important to note that purified bioactive substances and non‐biomass metabolites fall outside this definition [12].

The potential of postbiotics is evident across various applications. For instance, they have been successfully applied in the baking industry [13, 14], and emerging studies indicate that postbiotics may even outperform live probiotics in certain contexts, such as modulating gut microbiota in mouse models of colitis [15]. These findings prompt a reevaluation of whether the benefits traditionally ascribed to live probiotics are partly mediated by the bioactive components they produce.

The production of postbiotics involves the inactivation of microorganisms via physical, chemical, or biological methods—a process that critically influences the preservation of key bioactive components [16, 17, 18]. Beyond traditional health applications, postbiotics have demonstrated diverse benefits, including cholesterol reduction, immunomodulation, and behavioral effects noted in psychobiotic research [19, 20]. Their sources are varied, encompassing lactic acid bacteria (LAB), bifidobacteria, and yeast, with active components such as peptidoglycans, lipopolysaccharides (LPS), and lipoteichoic acids [21].

In this review, we critically examine the current status of postbiotics, focusing on their potential benefits, inherent challenges, and broader implications for advancing human and animal health. By elucidating the underlying mechanisms of both probiotic and postbiotic activities, we aim to highlight key research gaps and inform future strategies for their safe and effective application.

### 1.1 Postbiotics: An Existing Concept That Still Needs to Be Defined

Postbiotics are beginning to make significant strides in both fundamental and applied microbiological research, offering new perspectives on an established idea. The suggestion that nonliving microorganisms and/or their components may confer health benefits to consumers or patients (collectively referred to here as the “host”) carries far‐reaching implications for their application [17]. For example, the fermentation process used in preparing various fermented foods illustrates how starter and adjuvant cultures can have positive health effects even after the microbes have been inactivated [22].

In this context—and given the absence of a universally accepted definition of postbiotics—a natural question arises: are enzymes, antibiotics, bacteriocins, γ‐aminobutyric acid (GABA), and similar metabolites that are produced industrially and subsequently purified considered postbiotics? According to the definition proposed by the International Scientific Association for Probiotics and Prebiotics (ISAPP), such purified extracts are not classified as postbiotics because they are separated from the bacterial biomass and delivered to eukaryotic hosts in the form of extracts or partially purified preparations. However, can sterilized or pasteurized fermented food products be considered postbiotic products? Most likely, yes, since the beneficial metabolite(s) produced by bacterial cultures remain in the food matrix even after the producing cells have been inactivated.

The health‐promoting properties of postbiotics are linked to the specific mechanisms of action of various metabolites produced by beneficial microorganisms. Specific postbiotics can modulate immune responses, inhibit pathogen binding, maintain intestinal barriers, stimulate brain function, and aid in controlling carcinogenesis and infections. Moreover, postbiotics exhibit antimicrobial, antioxidant, and immunomodulatory properties, thereby benefiting multiple physiological, immunological, neurohormonal, regulatory, and metabolic processes within the host [23].

Compared with probiotics, postbiotics offer several advantages because they consist of inactivated microbes and their preformed metabolites. These advantages include the absence of risks associated with bacterial translocation from the intestines into the bloodstream and a reduced likelihood of acquiring antibiotic‐resistant genes. Additionally, postbiotics are easier to extract, formulate, standardize, transport, and store than living microorganisms [23, 24, 25]. Existing research highlights the promising potential of postbiotics in diverse fields such as food preservation, nutritional enhancement, cosmetics, medicine, and overall human health [23, 24, 25, 26].

## 2 Mechanisms of Action of Postbiotic Formulations on Targeted Eukaryotic Hosts

The mechanisms by which postbiotic preparations influence host microbiota and overall health remain incompletely understood, partly due to the heterogeneous nature of these formulations. It is important to emphasize that the mechanisms of action of postbiotics and probiotics can be similar in several aspects, as many of them are associated with specific metabolites produced by probiotics and present in postbiotics (ultimately, postbiotics are derived from living cells). Therefore, in the following discussion, we aim to explore how postbiotics provide their benefits while using probiotics as comparative points. Postbiotics may comprise non‐viable cells, cellular debris, short‐chain fatty acids (SCFAs), weak organic acids, bacteriocins, and other metabolites. Notably, the antimicrobial activity of postbiotics often persists following microbial inactivation. For example, lactic acid [27] and bacteriocins [28] retain direct antimicrobial effects, while *quorum quenching* molecules [29] can indirectly modulate microbial populations. In addition, if key surface molecules—such as fimbriae [30] or lectins [31]—remain intact post‐inactivation, postbiotic preparations may compete with resident microorganisms for adhesion sites.

Extracellular vesicles (EVs), also known as outer membrane vesicles (OMVs) in some bacterial species, are secreted by eukaryotes, prokaryotes, and archaea. These membrane‐derived lipid bilayers contain proteins critical for host‐microbe and microbe‐microbe communication and signaling [32]. EVs from probiotic or commensal bacteria (e.g., lactobacilli, *Akkermansia*, and *Pediococcus* spp.) are generally associated with beneficial molecule delivery, whereas those from pathogens typically transport harmful substances—such as LPS, peptidoglycans, nucleic acids, virulence factors, and toxins—that elicit specific host immune responses [33, 34]. The health implications of these EVs and their postbiotic components represent an exciting new research frontier.

Bacterial cells are often enveloped in EPS, which may appear as a tightly bound capsule or a loosely organized slime layer. These structures protect bacteria from host immune responses and adverse environmental conditions [35]. EPS have demonstrated a broad range of bioactivities—including antimicrobial, anti‐tumor, anti‐biofilm, anti‐viral, and immunomodulatory effects—along with the ability to reduce lipid peroxidation and deposition in vessels, enhance antioxidant enzyme activity, and lower blood pressure [36]. Additionally, EPS find diverse applications in biomedical fields (e.g., drug delivery systems and wound dressings) and the food industry (e.g., as stabilizers, emulsifiers, and agents for moisture retention) [35].

Commensal gut bacteria contribute a wide array of postbiotic molecules capable of influencing multiple host systems by modulating immune responses, hormone signaling, metabolic pathways, and both central and peripheral nervous system functions. Investigations into these interactions suggest that the majority of postbiotic effects are benign, modulatory, or beneficial. Moreover, commercial efforts are underway to identify postbiotic small molecules, assess their pharmacological activities, and develop novel patentable therapeutics [37]. Given that beneficial postbiotics have co‐evolved with healthy human hosts, they are hypothesized to exhibit a high probability of effectiveness and a broad margin of safety when applied therapeutically.

The following section highlights recent examples of beneficial postbiotic bacteria or bacterial components and the reported mechanisms of their action.

### 2.1 Enhancing Epithelial Barrier Function

Postbiotic formulations can support epithelial barrier integrity through a variety of mechanisms. Key bioactive components include secreted proteins such as Msp1/p75, Msp1/p40 [38], and HM0539 [39]. In addition, EPS produced by beneficial bacteria and retained in postbiotic preparations helps reinforce barrier function by reducing inflammation through specific signaling pathways [40]. Certain *Bifidobacterium* species have been reported to activate mitogen‐activated protein kinase signaling, thereby enhancing tight junction function via autophagy and calcium signaling [41]. Furthermore, SCFAs such as acetate, propionate, and butyrate improve transepithelial resistance and promote tight junction formation in intestinal epithelial cells [42].

Maintaining a functional gut barrier—selectively permeable to beneficial molecules while excluding harmful substances—is critical for host health. Impaired barrier function is associated with various disease states [43]. For instance, high‐fat‐diet (HFD)–fed mice treated with EVs derived from *Akkermansia muciniphila* showed reduced gut permeability (as evidenced by lower FITC‐dextran uptake), diminished inflammatory cell infiltration, and increased expression of tight junction proteins [44]. Similarly, EVs from *A. muciniphila* (but not from *Escherichia coli*) decreased LPS‐induced permeability in Caco‐2 monolayers via increased phosphorylation of AMPK—a kinase integral to tight junction assembly and stability [44]. The protection conferred by EVs is further supported by studies showing that *A. muciniphila* EVs ameliorated dextran sodium sulfate (DSS)‐induced colitis phenotypes, including body weight loss, reduced colon length, and inflammatory infiltration [45].

In addition, EVs derived from *E. coli* Nissle 1917 enhanced transepithelial resistance and elevated the expression of several tight junction proteins in polarized T‐84 and Caco‐2 monolayers [46]. This mechanistic insight supports clinical observations of remission induction in ulcerative colitis patients treated with *E. coli* Nissle 1917 [47, 48]. Furthermore, in vitro studies using cell‐free extracts or supernatants from lactobacilli have demonstrated anti‐inflammatory, antioxidant, and barrier‐protective effects [49, 50, 51, 52]. Notably, HM0539—a protein purified from *Lacticaseibacillus rhamnosus* GG culture supernatant—enhanced gut barrier integrity in Caco‐2 cells by upregulating mucins and tight junction proteins and provided protection against *E. coli* K1 pathogenesis in neonatal rats, DSS‐induced colitis in mice, and LPS/D‐galactosamine‐induced bacterial translocation and liver injury in mice [53]. Within the gut, EPS also contribute by reducing bacterial adhesion to the intestinal epithelium and by modulating host immune responses via interactions with Toll‐like receptors (TLRs) and C‐type lectin receptors (CLRs) [54].

### 2.2 Immunomodulatory Activities

Postbiotics can modulate immune responses through microorganism‐associated molecular patterns (MAMPs) that engage pattern recognition receptors (PRRs) such as Toll‐like receptors (TLRs), NOD‐like receptors, and C‐type lectins. These interactions trigger cytokine production and broader immune modulation [55]. For example, lipoteichoic acid interacts with TLR2/6 [56], peptidoglycan‐derived muropeptides activate NOD2 [57], and CpG‐DNA stimulates TLR9 [58]. Such interactions are likely preserved in postbiotic preparations if the effector molecules remain intact following inactivation. In addition, immunostimulatory lysates from both Gram‐positive and Gram‐negative bacteria have been shown to activate TLR4 and TLR2 [59]. Moreover, metabolites like lactic acid mediate immune responses via GPR31, promoting the formation of intestinal dendrite protrusions [60].

### 2.3 Systemic Metabolic Responses

Postbiotics can modulate systemic metabolism through a range of metabolites and enzymes derived from inactivated microorganisms. For example, bile salt hydrolase deconjugates bile acids, facilitating microbial biotransformation that diversifies the circulating bile acid pool and thereby influences glucose, lipid, and energy metabolism [61]. Additionally, succinate—an intermediate produced during carbohydrate fermentation—enhances intestinal gluconeogenesis and glycemic control [62]. Furthermore, SCFAs contribute to metabolic homeostasis; propionate improves insulin sensitivity, while butyrate upregulates antioxidant defenses through increased glutathione production [63].

*A. muciniphila*, a Gram‐negative microaerophilic bacterium specialized in mucin degradation, has emerged as a key player in cardiometabolic regulation [64, 65]. In animal models, increasing the relative abundance of *A. muciniphila*—either through dietary polyphenol supplementation [66, 67, 68, 69, 70, 71, 72, 73, 74] or by oral administration of live bacteria [75, 76, 77]—has been associated with reduced cardiometabolic symptoms. Notably, heat‐killed *A. muciniphila* (prepared at 70°C for 30 min) that retains its cell surface protein structures has been shown to be as effective, or even more so, than live bacteria in preventing adiposity, glucose intolerance, insulin resistance, and dyslipidemia in HFD‐fed mice [75]. These benefits have also been observed in humans; individuals with overweight/obesity and insulin resistance experienced improved insulin sensitivity after 3 months of consuming heat‐inactivated *A. muciniphila*, with no major shifts in overall microbial community structure [78].

Several *A. muciniphila* components and metabolites contribute to its metabolic benefits. Gentle pasteurization is thought to enhance the exposure of Amuc_1100—a 33 kDa outer membrane protein (also known as the pilus‐associated signaling (PAS) protein) [75]. In rodent studies, oral administration of purified Amuc_1100 attenuated HFD‐induced metabolic disturbances by reducing body weight gain, lowering plasma LPS levels (indicative of improved gut barrier function), increasing the expression of jejunal tight junction proteins, and improving glucose tolerance [75, 79, 80]. Amuc_1100 directly interacts with Toll‐like receptor 2 (TLR2), a receptor implicated in the regulation of epithelial barrier integrity [75, 81].

Another significant component is Amuc_1631, also known as P9, an 84 kDa protein secreted by *A. muciniphila*. P9 binds directly to intercellular adhesion molecule 2 (ICAM‐2), an immune cell integrin critical for cell barrier penetration [82]. In HFD‐fed mice, purified P9 reduced weight gain, improved glucose tolerance, and increased the expression of uncoupling protein 1 (UCP‐1) in brown adipose tissue, suggesting the induction of non‐shivering thermogenesis. P9 also acts as a glucagon‐like peptide‐1 (GLP‐1) agonist; treatment of human L‐cells (NCI‐H716) or primary intestinal epithelial cells resulted in increased GLP‐1 secretion. Notably, the metabolic benefits of P9 were abrogated in IL‐6 knockout mice, indicating that its bioactivity requires IL‐6 [82].

In addition to protein effectors, membrane lipids can contribute to systemic metabolic responses. An immunomodulatory diacyl phosphatidylethanolamine with two branched chains (a15:0‐i15:0 PE), which constitutes approximately 50% of the *A. muciniphila* lipid membrane, acts as an agonist for non‐canonical TLR2–TLR1 heterodimers in human monocytes and dendritic cells. This interaction promotes the selective release of inflammatory cytokines and is thought to reset immune activation thresholds, thereby supporting homeostatic immunity [83].

Beyond these direct effects, peptidoglycan from bacterial cell walls can translocate from the gut to the bone marrow, where it primes neutrophils via nucleotide‐binding oligomerization domain‐containing protein‐1 (Nod1). Neutrophils primed in this manner exhibit enhanced pathogen‐killing capacity; however, this effect is markedly reduced in Nod1 knockout or germ‐free mice [84].

The liver is also a key target for postbiotic action. Metabolic‐associated steatotic liver disease (MASLD), formerly known as nonalcoholic fatty liver disease (NAFLD), can progress from hepatic steatosis to non‐alcoholic steatohepatitis (NASH), cirrhosis, and even hepatocellular carcinoma [85]. In metabolic disease, EVs may transport pro‐inflammatory bacterial components to the liver via the portal vein, exacerbating liver inflammation [85]. However, postbiotics offer therapeutic potential for MASLD. For instance, oral administration of EVs from *A. muciniphila* supernatants reduced liver fibrosis markers in HFD‐fed mice treated with carbon tetrachloride (CCl_4_), a model of hepatic fibrosis [86]. Similarly, SCFAs supplementation alleviated hepatic steatosis and inflammation in mice fed a methionine‐ and choline‐deficient diet [87]. In addition, bacterial metabolites such as indole‐3‐acetic acid (IAA) and indole‐3‐propionic acid (IPA) have shown promise in reducing hepatic oxidative stress, inflammation, and triglyceride accumulation in HFD‐fed rodents, with lower circulating IPA levels correlating with liver fibrosis in humans [88, 89, 90, 91].

Taken together, these findings underscore the diverse mechanisms by which postbiotic components influence systemic metabolism and highlight their potential in preventing and treating metabolic disorders. Further investigation into these postbiotic agents is warranted, particularly given the current lack of effective therapies for MASLD.

### 2.4 Neuroactive Effects

Postbiotic preparations can generate neuroactive compounds that influence behavior and cognitive function [92]. These formulations may contain neurotransmitters—including serotonin, dopamine, acetylcholine, and GABA—as well as bioactive compounds such as indoles and bile acids that interact with brain receptors. In addition, microbial enzymes convert dietary precursors (e.g., tryptophan for serotonin and tyrosine for dopamine), thereby modulating neurotransmitter synthesis and availability [93].

The gut‐brain axis facilitates bidirectional communication between gut microbes and the central nervous system (CNS), allowing microbial products to impact cognitive health. For instance, the *A. muciniphila* protein Amuc_1100 (purified via recombinant expression in *E. coli*) has been shown to improve depression‐like behaviors in a murine model of chronic unpredictable mild stress (CUMS) by elevating serum and colonic serotonin levels, increasing brain‐derived neurotrophic factor (BDNF), and dampening neuroinflammation [94]. A truncated version of this protein, Amuc_1100Δ80, which lacks the first 80 N‐terminal amino acids, exhibits a higher affinity for TLR2 and more effectively reduces anxiety and depressive behaviors in stressed mice [95]. In another study, Amuc_1100 restored BDNF and tropomyosin receptor kinase B (TrkB) expression in the hippocampus and cortex, alleviating anxiety and depression induced by an antibiotic cocktail [96].

Moreover, many gut bacteria (primarily *Bacteroides*) and common probiotics (notably lactobacilli and bifidobacteria) encode glutamic acid decarboxylase (GAD), an enzyme that converts glutamate to GABA. This inhibitory neurotransmitter is critical for cognitive function and is often diminished in individuals with metabolic or neurological disorders [97]. Additionally, bacterial metabolites derived from dietary polyphenols—such as urolithin A (a metabolite of ellagic acid), dihydroxyphenyl‐γ‐valerolactones (from flavan‐3‐ols and proanthocyanidins), and equol (from isoflavones)—have demonstrated neuroprotective effects [98].

### 2.5 Anti‐Inflammatory Activity

Postbiotic components from probiotic strains and commensal microbes can modulate the host immune system to achieve mutual benefit. For example, cell‐free supernatants (CFS) from *Lactobacillus acidophilus* and *Lacticaseibacillus casei* have been shown to reduce the secretion of pro‐inflammatory cytokines (such as TNF‐α) while boosting anti‐inflammatory cytokine (e.g., IL‐10) production in intestinal epithelial cells. These supernatants also exhibit antioxidant activity, reducing oxidative stress in vivo [99, 100, 101]. Similarly, CFS from *Lactiplantibacillus plantarum* has been reported to enhance the absorptive surface of the intestine and reduce the load of intestinal pathogens in lambs [102].

*A. muciniphila* is recognized as a significant producer of ornithine lipids in both human and murine guts; these lipids can be detected in EVs produced in vitro [103]. Ornithine lipids play an immunoregulatory role by preventing LPS‐induced inflammation in bone marrow‐derived macrophages. They achieve this by upregulating Atf3, a transcription factor that dampens TLR4 activation and reduces the expression of pro‐inflammatory cytokines (including IL‐1β, MCP‐1, MIP‐1α, GM‐CSF, IL‐12, and RANTES) [103]. Furthermore, EVs derived from *Lpb. plantarum* promote the differentiation of human monocytic THP1 cells toward an anti‐inflammatory M2 phenotype and foster an anti‐inflammatory cytokine profile in cultured human skin tissue, suggesting potential applications for alleviating hyperinflammatory skin conditions [104].

### 2.6 Anticancer Activity

Bacteriocins present in postbiotic preparations can selectively bind to the negatively charged membranes of cancer cells, inducing cell death without harming normal cells. For example, plantaricin BM‐1 from *Lpb. plantarum* induces apoptosis through caspase‐dependent pathways and downregulates genes involved in TNF and MAPK signaling in SW480 colorectal cancer cell lines [105]. Similarly, nisin produced by *Lactococcus lactis* exhibits cytotoxic activity against astrocytoma, neuroblastoma, and colorectal cancer cell lines, while nisin Z has shown efficacy against human malignant melanoma cells [106].

In contrast, extracellular vesicles (EVs) from oral *Fusobacterium nucleatum*, a bacterium implicated in colorectal cancer (CRC), carry pro‐inflammatory and proliferative mediators that can promote tumorigenesis [107]. Notably, cell‐free extracts from *Bifidobacterium longum* have been found to inhibit the proliferation, migration, and invasion of CRC cells exposed to *F. nucleatum* EVs by suppressing certain oncogenes [108]. However, these extracts may also upregulate some oncogenes and shift cellular metabolism toward anaerobic glycolysis, potentially favoring CRC progression. This duality underscores the need for careful characterization of postbiotic components [108].

Recent reviews have highlighted the promising potential of postbiotics in cancer therapy. Various components—including cell‐free extracts, EPS, SCFAs, conjugated linoleic acids, and peptidoglycans—from probiotic strains (such as representatives from lactobacilli, *Pediococcus* spp., bifidobacteria, *Bacillus* spp., and *Propionibacterium* spp.) have been shown to inhibit cancer cell growth via anti‐inflammatory, apoptotic, and antiproliferative mechanisms [106].

### 2.7 Effects of Postbiotics on In Utero and Infant Development

Recent studies indicate that the influence of postbiotics on development begins in utero. Although live bacteria are generally excluded from the intrauterine environment, a range of postbiotic molecules derived from the maternal microbiota can cross the placental barrier [109, 110]. For example, in a gestation‐only colonization model, germ‐free (GF) pregnant mice were transiently colonized with *E. coli* HA107—a strain auxotrophic for certain amino acids and unable to persist in the mouse gut. Although the bacteria survived only for 24–72 h, pups born to these transiently colonized dams exhibited notable immune modifications. Compared to GF controls, these pups showed increased numbers of class 3 innate lymphoid cells (ILC3) and mononuclear cells in the intestine for up to 60 days postnatally, while B and T cell populations in systemic organs remained unchanged [111]. RNA sequencing of the small intestinal mucosa from 14‐day‐old neonates further revealed the upregulation of genes involved in antimicrobial peptide production, sugar metabolism, cell division, differentiation, ion transport, mucus secretion, and xenobiotic as well as bile acid metabolism. Moreover, experiments using radiolabeled *E. coli* HA107 demonstrated that a diverse array of bacterial metabolites was transferred from the mother to the offspring between postnatal Days 1 and 11. These metabolites, including ligands for the aryl hydrocarbon receptor (AhR), are known to promote ILC3 expansion and enhance pathogen resistance [111, 112, 113]. Stable isotope tracing further confirmed that, within hours after gavage with labeled *E. coli* HA107, microbial metabolites belonging to various compound classes (amino acids, lipids, carbohydrates, vitamins, pigments, aromatic compounds, steroids, etc.) could be detected across multiple host organs, including the brain, gastrointestinal tract, bone marrow, muscle, fat, liver, spleen, pancreas, kidney, thymus, and lymph nodes [114]. Collectively, these findings suggest that intrauterine exposure to postbiotics primes the offspring's immune system and other organ systems for subsequent colonization at birth.

The benefits of postbiotics extend into the postnatal period. In a study with female Wistar rats, maternal supplementation with *Lbs. rhamnosus* GG during a hypercaloric diet resulted in offspring with lower food intake, normalized birth weights, improved glucose sensitivity, and reduced mesenteric adiposity compared with controls [115]. Although the precise mechanisms underlying these positive metabolic outcomes were not fully elucidated, postbiotic‐mediated conditioning of the developing immune and metabolic systems is a likely contributor. After birth, postbiotic molecules continue to be transferred to the neonate through breast milk, alongside secretory IgA, human milk oligosaccharides (HMOs), and a variety of commensal bacteria, including bifidobacteria, lactobacilli, and staphylococci species [110, 116]. The use of fermented infant formulas—produced by fermenting milk with specific microbial strains followed by heat inactivation—has shown promise in replicating some of the benefits of breast milk. Infants receiving these postbiotic‐rich formulas exhibit gut microbial diversity, secretory IgA levels, and metabolomic profiles more closely resembling those of breastfed infants, along with fewer atopic, digestive, and respiratory symptoms compared to those fed non‐fermented formulas [116, 117, 118, 119]. Moreover, randomized controlled trials have demonstrated that fermented cow's or rice milk containing heat‐killed *Lacticaseibacillus paracasei* CBA L74 reduces the incidence of upper respiratory and acute gastrointestinal infections in young children (12–48 months). Follow‐up analyses revealed that this intervention promoted the growth of butyrate‐producing bacteria in the gut, thereby enhancing both innate and acquired immunity [120, 121].

In summary, postbiotic exposure in utero and during early infancy appears to play a critical role in shaping the immune system, metabolic regulation, and overall health of the developing organism. Further research is warranted to optimize strain selection, combinations, and the bioactivity profiles of postbiotic preparations to maximize these benefits.

### 2.8 Antimicrobial Activity: Bacteriocins

In vivo studies, particularly those involving various lactobacilli strains, have demonstrated that postbiotic derivatives from lactobacilli are a rich source of bacteriocins, which restrict the growth and activity of diverse pathogens [122].

Bacteriocins are relatively small, predominantly cationic, genetically encoded proteinaceous antimicrobial compounds produced by virtually all bacteria. They can exhibit either narrow‐spectrum activity—targeting the same species—or broad‐spectrum activity—affecting multiple genera—and are often involved in *quorum sensing* and *quorum quenching* processes [123]. While broad‐spectrum bacteriocins are effective against a wide array of pathogens, narrow host‐range bacteriocins can target specific pathogens without disrupting the normal microbiota, making them promising candidates for precision medicine in the treatment of infectious diseases [124]. Typically, their bactericidal action is mediated by pore formation in the cytoplasmic membrane, and they rarely display cross‐resistance with conventional antibiotics [125].

Originally considered solely as antimicrobial agents, bacteriocins have since been recognized for their role in enhancing the probiotic potential of their producer strains. They contribute to microbiota modulation, immune system regulation, and improved colonization of probiotics on host surfaces [126]. More recently, bacteriocins have been discussed as effective microbiome‐editing tools for correcting dysbiosis and shaping the host microbiome [124]. These properties underscore their suitability for incorporation into postbiotic preparations.

Among the antimicrobial components present in postbiotic formulations, bacteriocins are notably robust and stable under the conditions used for microbial cell inactivation [125]. Many bacteriocins retain activity over a broad range of pH values and temperatures, even surviving autoclaving conditions (15 min at 121°C) [127]. Although the precise mechanisms underlying the overall antimicrobial activity of postbiotics remain to be fully elucidated, it is clear that bacteriocins play a critical role in preventing the growth of pathogenic bacteria and thereby contribute to host health [128]. To illustrate their importance, consider several examples of bacteriocin activity against pathogens. Curvacin A and sakacin 1, produced by *Latilactobacillus sakei* subsp. *sakei*, are active against *Listeria monocytogenes*. Plantaricin MG, produced by *Lpb. plantarum* subsp. *plantarum*, inhibits the Gram‐negative pathogen *Salmonella typhimurium* as well as several Gram‐positive bacteria [129]. Gassericins A and T from *Lactobacillus gasseri* inhibit *L. monocytogenes*, *Staphylococcus aureus, and Bacillus cereus*, while the antilisterial bacteriocin ABP‐118 from *Ligilactobacillus salivarius* is active against *Listeria* spp.*, Bacillus* spp.*, Staphylococcus* spp.*, and Salmonella* spp. (for review, see Scott et al. [130]). Additionally, nisin produced by *L. lactis* demonstrates both antibiofilm and antimicrobial activity against several Gram‐positive foodborne and oral pathogens such as *Porphyromonas gingivalis, Fusobacterium nucleatum*, and *Prevotella intermedia* [131]. Collectively, these reports suggest that postbiotic preparations enriched with bacteriocins hold significant potential as potent antimicrobials for food preservation [132] and for the prevention and treatment of infections—with possibly fewer side effects than conventional antibiotics. Moreover, the antimicrobial potency of bacteriocins can be further enhanced by specific synbiotic sugars. For instance, Harold et al. [133] demonstrated that the addition of 0.5% (w/v) galactose and 2.5% (w/v) raffinose significantly stimulated lantibiotic gene expression in *Streptococcus salivarius* BLIS K12 and BLIS M18, respectively, thereby broadening their bacterial inhibition range and potency.

Most of the studies discussed above have focused on bacteriocins that were purified, semi‐purified, or obtained from crude cell‐free supernatants of probiotic cultures. To our knowledge, no studies have yet reported on bacteriocins present in postbiotic preparations generated solely by the inactivation of microbial cells without subsequent purification. Given the diverse applications of bacteriocins, it is reasonable to anticipate that these molecules can retain their antimicrobial properties in postbiotic formulations, thereby enhancing the safety of products intended for human consumption (for review, see Liang and Xing [134]) and contributing to overall host health.

## 3 Postbiotic Formulations on Targeted Microbial Cells

The antimicrobial activity of heat‐killed microbial preparations has been widely demonstrated in both in vitro and in vivo studies. These postbiotic formulations—derived from heat‐killed cell lysates—contain a diverse array of antimicrobial compounds, including bacteriocins, enzymes, small molecules, organic acids, and other bioactive substances that act against both Gram‐positive and Gram‐negative bacteria [128]. Although the precise mechanisms underlying their antimicrobial effects remain to be fully elucidated, postbiotics generally improve host health by inhibiting the growth of pathogenic bacteria.

For example, in a murine model, a heat‐killed multispecies LAB formulation effectively reduced *Salmonella* invasion and inflammation mediated by lipoteichoic acids and exopolysaccharides [135]. Similarly, heat‐killed *Lactobacillus johnsonii* inhibited the growth of *Helicobacter pylori* in vitro, inducing notable cellular deformations—such as loss of the spiral shape, bending of the cell body, and eventual degradation [136]. In another instance, heat‐inactivated *Bifidobacterium animalis* subsp. *lactis* BB12 interfered with *Streptococcus mutans* biofilm formation in dentinal cavities [137].

Several studies have also evaluated the in vitro activity of various postbiotic preparations. For instance, the cell‐free supernatant (CFS) of *B. longum* ATCC15707, containing 22.7 mg/mL of total proteins and 7.3 mg/mL of total fatty acids, demonstrated bactericidal effects at a dose of 40 mg/mL by nearly completely inhibiting *Enterobacter cloacae* and reducing intraluminal pH [122]. Similarly, postbiotics derived from *Lab. acidophilus* strains NCC 2581, NCC 2538, and NCC 2592 inhibited the growth of *Giardia lamblia* in vitro and prevented cyst formation [138].

It is noteworthy that many investigators refer to cell‐free supernatants and partially purified bioactive preparations of microbial origin as “postbiotics” (for reviews, see Liang and Xing [134] and Ramazanidoroh et al. [139]). While this usage does not strictly adhere to the definition of postbiotics proposed by the ISAPP, it remains important to critically discuss these studies. Despite not fully meeting the ISAPP definition, these formulations frequently contain bacteriocins that control the growth and activity of undesirable microorganisms, including both pathogenic and spoilage bacteria.

## 4 Methods of Inactivation of Live Microorganisms to Generate Inanimate Postbiotic Formulations

**FIGURE 2:**
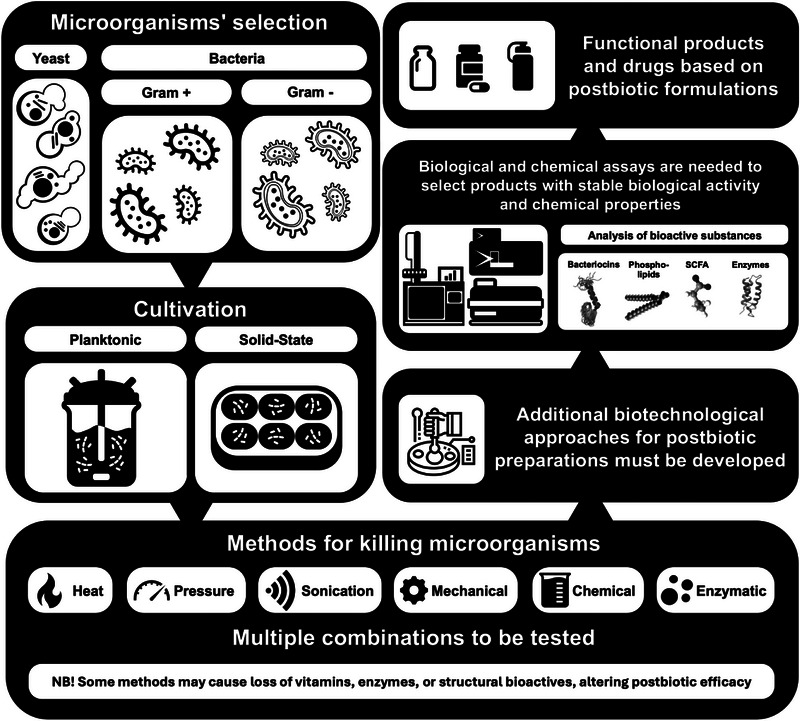
The concept of creating postbiotics: selection of microorganisms, scientific substantiation of beneficial properties, optimization of biotechnological production and development of recipes for use.

The development of effective postbiotics and their associated health benefits requires careful attention to several factors, including the accurate genetic identification of the microorganisms, the selection of appropriate inactivation procedures, and the quantification of the final postbiotic composition (Figure 2). The microorganisms employed for postbiotic preparation need not be probiotics per se; rather, their genetic identity must be well characterized. Equally critical is the choice of physico‐chemical techniques used for microbial inactivation. Both thermal and non‐thermal methods have been applied, and—as noted in a comprehensive review by Shehadul et al. [140]—the selection of the inactivation method plays a vital role in the performance of the resulting postbiotic formulations.

### 4.1 Heat Inactivation

Thermal processing, long used in the food industry to ensure enzymatic and microbiological stability [141], is the most universal method for preparing postbiotics. This approach includes techniques such as pasteurization, sterilization, ohmic heating, radiofrequency heating, and microwave heating. Although these methods primarily rely on thermal effects for microbial inactivation, their effectiveness varies with the mode of heat generation [142].

For example, convection—used in boiling or circulating hot air—transfers heat through fluid movement, whereas ohmic heating generates heat directly within the material by passing an electrical current, allowing for rapid and even heating. Both methods are widely used in agriculture and food processing [143]. Ohmic heating, in particular, is emerging as a promising technology not only for food processing but also for the valorization of bioresources such as agri‐food waste, forestry surplus, seaweed, and microalgae [144]. Its efficiency depends on several parameters—including temperature, electric field strength, frequency (data on which is still limited), and the composition of the treated matrix—and may even exert non‐thermal effects within certain temperature ranges. Some studies suggest that foods subjected to ohmic processing have lower D‐values (i.e., require less time for microbial inactivation) than those treated by conventional methods, especially under conditions of increased electric field strength, higher salt content, and lower pH, fat, and solids content (for review, see Muller et al. [145]).

Heat inactivation is widely used both for eliminating microorganisms and spoilage agents in food safety and for generating postbiotics. Some authors have even reported the formation of “nanoscale postbiotics” after heat treatment—a significant advancement for applications in food and pharmaceutical industries [146]. However, high‐temperature processing (e.g., autoclaving at 121°C) may inactivate some postbiotic functions, whereas milder thermal treatments (below 100°C) have been shown to enhance antioxidant and anti‐inflammatory properties compared to live probiotics [96].

Traditional thermal inactivation has been described in numerous studies—ranging from in vitro experiments to clinical trials—demonstrating beneficial effects in gastrointestinal modulation, bone loss prevention, antimicrobial activity, endocrine health promotion, and improvements in the gut‐brain axis. For instance, a heat‐inactivated preparation of *Lactobacillus helveticus* improved bowel movements, gastrointestinal function, and mood in healthy subjects, suggesting its potential as a “psychobiotic” [147]. Similarly, a randomized, placebo‐controlled, double‐blind study of a heat‐killed postbiotic derived from *Lpb. plantarum* demonstrated immunomodulatory effects in individuals with normal white blood cell counts [148]. Furthermore, postbiotics from heat‐killed probiotics have been shown to act similarly to live *B. longum* cells by significantly reducing symptoms of diarrhea‐predominant irritable bowel syndrome (IBS‐D) [149]. Notably, the effectiveness of both pro‐ and postbiotics depends on the microbial species or strain, the target host, and the specificity of their interactions. In a mouse model of Alzheimer's disease, for example, both live and heat‐inactivated *Streptococcus thermophilus* cells modulated the gut‐brain axis to alleviate cognitive dysfunction, albeit through different mechanisms [29]. Additionally, studies in model organisms such as *Caenorhabditis elegans* have reported lifespan extension following postbiotic treatment [150, 151, 152].

### 4.2 Thermal Pasteurization

Thermal pasteurization is a milder form of heat treatment designed to inactivate relatively heat‐sensitive microorganisms, such as vegetative bacteria, yeasts, and molds responsible for food spoilage and foodborne illnesses [153]. In applications like fruit juice processing, pasteurization typically involves heating temperatures between 60°C and 100°C to reduce microbial loads or deactivate enzymes, though spores are generally unaffected. The efficacy of pasteurization is quantified by the reduction in target microorganism populations or enzyme activities—termed “lethality.” Depending on the intensity of the heat treatment, pasteurization can be categorized as high‐temperature long time (HTLT), high‐temperature short time (HTST), mild‐temperature‐long time (MTLT), or mild‐temperature‐short time (MTST) [154].

It is important to note that even “mild” heat treatments can affect the bioavailability of bioactive molecules (e.g., vitamins, polysaccharides, enzymes, proteins) or lead to the formation of toxic substances. These nuances necessitate a careful balance between microbial inactivation and the preservation of beneficial postbiotic properties.

### 4.3 Non‐Thermal Inactivation of Microbial Cells

Thermal inactivation can irreversibly damage thermosensitive bioactive molecules—particularly proteins and polypeptides—in microbial cells. Consequently, non‐thermal inactivation methods are gaining attention for preserving these functional components [142]. Non‐thermal techniques include the application of electric fields, ultrasonication, high pressure, x‐rays, ionizing radiation, high‐voltage electrical discharge, pulsed light, and various forms of magnetic field heating, as well as plasma technology [141, 155].

Spray drying, which rapidly dries a liquid or slurry with hot gas to produce a dry powder, has been proposed as a cost‐effective alternative to freeze drying for creating dehydrated, inactivated microbial cultures. Other methods, such as vacuum drying and fluidized bed drying, can also stress microorganisms enough to reduce viability, sometimes leading to complete inactivation [156]. Often, combining or sequentially applying these milder technologies—possibly along with moderate temperature treatments—yields greater microbial inactivation [157]. The degree of inactivation, however, is influenced by factors such as cell type (e.g., prokaryotes vs. eukaryotes, Gram‐positive vs. Gram‐negative, vegetative cells vs. spores, cocci vs. rod‐shaped organisms), processing conditions, and composition of the surrounding matrix [158].

### 4.4 Inactivation by Ultrasound

Ultrasound uses sound waves with frequencies above 20 kHz to inactivate microorganisms. Its primary mechanisms include disrupting cytoplasmic membranes and generating free radicals [159]. In food processing, ultrasound is used to extend shelf life by reducing microbial loads while preserving sensory qualities such as taste, texture, and nutritional value [160]. Although ultrasound typically achieves only a 1–2 log reduction in microbial counts after 15–20 min of treatment, its potential can be enhanced when combined with other stresses. For example, ultrasound‐inactivated *Lbs. casei* has been shown to improve gut health and reduce cardiovascular damage in high‐calorie diet rat models [161]. Similarly, ultrasound‐treated *B. longum* SPM1207 lowered cholesterol levels, reduced body weight, and alleviated constipation in rats on high‐cholesterol diets [162].

Gram‐negative bacteria are generally more sensitive to ultrasound than Gram‐positive bacteria [163]. Factors such as cell wall composition, sonication temperature, and duration influence the efficiency of microbial inactivation [164]. Thermosonication—combining moderate heat with ultrasound—has been particularly effective at inactivating bacterial spores at temperatures below 90°C [165]. Moreover, sonication‐lysed preparations of *Bacillus velezensis* Kh2‐2 have demonstrated immunomodulatory effects in vitro, *ex vivo*, and in vivo, with postbiotics produced via sonication showing promising immunostimulatory and anticancer activities [83, 166, 167].

### 4.5 Inactivation by Ionizing Radiation

Ionizing radiation methods—including *γ*‐rays, high‐speed electrons, and x‐rays—inactivate microbial cells by damaging nucleic acids and generating oxidative stress via water ionization [168]. Gamma irradiation below 10 kGy (typically from Co60 sources) is considered safe, with minimal toxicity and nutrient loss [169]. This method has been used to produce postbiotics from microorganisms such as *Lab. acidophilus, Limosilactobacillus reuteri, B. animalis, and Bifidobacterium lactis* [170, 171, 172]. For instance, inactivated *B. animalis* has been shown to lower blood glucose and cholesterol levels while modulating gut microbiota composition [171], and *B. lactis* has similarly reduced cholesterol levels in Wistar rats [172]. Additionally, *L. reuteri* has been reported to alleviate visceral pain in rat models [170].

### 4.6 Inactivation by Ultraviolet Light

Ultraviolet (UV) light is an economical, non‐thermal inactivation technology that is classified into UV‐A (320–400 nm), UV‐B (280–320 nm), and UV‐C (200–280 nm), with UV‐C being the most effective for bactericidal treatment. The advances in deep UV‐based inactivation (∼220 nm) have demonstrated enhanced microbial inactivation via oxidative damage to lipids and proteins [173]. UV light disrupts DNA transcription and translation, damages cell membranes, and impairs other cellular components. Its cost‐effectiveness and minimal impact on food properties make UV treatment a popular choice for inactivating pathogens in fruit juices, milk, and dairy products [174]. UV treatment has also been applied in postbiotic preparation; for example, UV radiation has been used to inactivate *Bacillus amyloliquefacien*s FPTB16 and *Bacillus subtilis* FPTB13—both of which exhibit immunomodulatory effects [170]. In addition, UV‐inactivated *Lbs. rhamnosus* GG has been shown to reduce IL‐8 levels in intestinal epithelial cells exposed to pathogenic ligands and flagellin [175].

## 5 Combination of Chemical and/or Physical Methods of Microbial Inactivation to Produce Health‐Modulating Formulations

Hybrid inactivation technologies that combine chemical and physical methods offer significant potential for producing postbiotics with preserved bioactivity. By integrating multiple techniques, these approaches may more effectively disrupt microbial cells while maintaining the integrity of sensitive bioactive compounds. For example, Tiptiri‐Kourpeti et al. [176] subjected *Lbs. casei* ATCC 393 to a sequential treatment—thermal processing at 100°C for 40 min, followed by ultrasound at 50 W for 10 min, and subsequent centrifugation at 13,000 × *g* for 40 min. This process yielded a postbiotic fraction that exhibited antiproliferative effects on cancer cells. Similarly, Amaretti et al. [101] treated cell suspensions of various bifidobacteria, lactobacilli, lactococci, and streptococci with ultrasound in five 1 min bursts at 0°C, followed by centrifugation to remove cellular debris; the resulting acellular supernatants displayed significant antioxidant potential. In another study, Choi et al. [177] and Dinić et al. [178] prepared fractions from heat‐killed lactobacilli by using ultrasound in a cooled water bath at 4°C, followed by centrifugation and ultrafiltration. The soluble polysaccharide fractions isolated from *Lab. acidophilus* 606 exhibited promising anticancer activity.

Dinic et al. [178] obtained a bioactive lysate from *Lmb. fermentum* BGHV110 through a combination of granulation (600 g for 10 min), high‐pressure homogenization in a French press (three passes), and freeze‐drying. This lysate demonstrated protective effects against acetaminophen‐induced hepatotoxicity in HepG2 cells. Likewise, Nakamura et al. [179] treated freeze‐dried cells from multiple strains (including *Bifidobacterium, Enterococcus, Issatchenkia*, lactobacilli, *Lactococcus, Leuconostoc*, and *Saccharomyces*) with a 0.5 M ethanolic solution of potassium hydroxide, followed by ultrasound treatment (300 W for 2 min), thermal treatment (boiling for 1 h), and solvent extraction using diethyl ether. The resulting fragmented cells were investigated as functional postbiotics for treating dyslipidemia in an obese mouse model. Aguilar‐Toala et al. [180] used a combination of ultrasound (42 kHz for 30 min) and enzymatic treatment (lysozyme/mutanolysin at 1 mg/mL, 37°C for 150 min) to obtain postbiotics from *Lbs. casei* CRL 431. Their bioactivity and multifunctional properties were assessed through global metabolite profiling using Raman spectroscopy. Similarly, Lee et al. [181] prepared intracellular extracts from *Lpb. plantarum* by incubating the strains with 1 mg/mL lysozyme at 37°C for 30 min, followed by ultrasonic disruption in five 1‐min intervals and centrifugation; these extracts exhibited potent antioxidant properties.

Despite these promising results, scalability remains a challenge. At industrial levels, changes in processing conditions can affect the stability and biological activity of postbiotic formulations. Additional research is required to refine extraction protocols and optimize cultivation conditions to facilitate large‐scale production [182].

A comparative analysis of various inactivation methods—both individually and in combination—was reported by Stănciuc et al. [183]. They evaluated traditional heat treatment, ohmic processing, high pressure, and ultrasound, along with combinations of these techniques, using *Lpb. plantarum* MIUG BL21 as a model. Their findings indicated that non‐viable cell suspensions exhibited cytocompatibility and promoted cell proliferation at higher dilution ratios. Moreover, lactate dehydrogenase release assays suggested potential tumor‐suppressing effects for samples treated with a combination of high pressure, ultrasound, and heat at the highest tested concentrations. The study emphasized that the biological activities of postbiotic formulations are significantly influenced by both the type and intensity of the inactivation methods employed.

The integration of mechanical, chemical, and enzymatic methods represents a comprehensive strategy for cell disruption. Mechanical methods such as bead milling, ultrasonication, and high‐pressure homogenization apply physical forces that break down cell walls and membranes, though they can generate heat and shear forces that may denature sensitive biomolecules. Chemical methods, which use detergents, solvents, and alkaline treatments, facilitate cell lysis by disrupting membranes and denaturing proteins; however, these agents may introduce contaminants if not carefully managed [35, 184]. Enzymatic treatments—with agents like lysozyme and proteases—offer high specificity in degrading cell wall components and are often used in combination with mechanical and chemical methods to enhance overall cell disruption efficiency [185, 186]. By synergistically combining these approaches, researchers can maximize cell lysis efficiency while minimizing damage to the desired bioactive components—a critical consideration for bioprocessing and biotechnology applications.

## 6 Probiotics vs. Postbiotics: Is There a Winner Yet?

**FIGURE 3:**
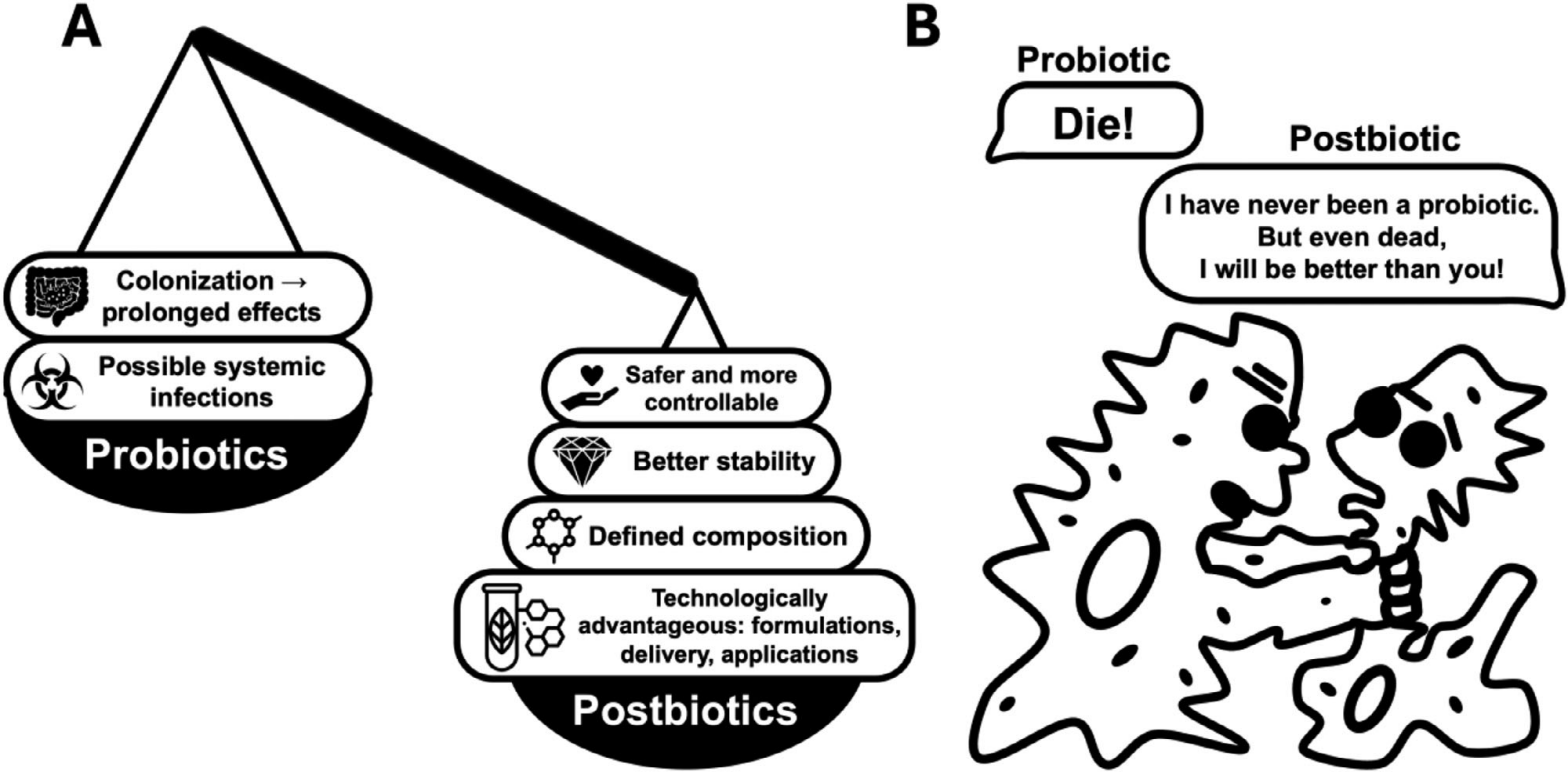
The pros and cons of using probiotics and postbiotics. Taking it a step further, or how the benefits of dead cells and expressed metabolites can be translated into health‐promoting properties.

The comparison of live versus killed cells in terms of their biological activity has been an area of increasing interest. Since the late 1990s, various health‐benefiting activities of inactivated and partially purified formulations derived from probiotics have been reported (for review, see Adams [187]). In this review, the author described the observation as paradoxical, separating the activities of live and dead probiotics. However, Adams [187] reached the critical conclusion that compared to probiotics, formulations with dead cells are likely to have a longer shelf life and are inherently safer. Another important point raised by Adams [187] is the necessity for establishing proper biomarkers for scientifically sound evaluations of the beneficial effects of both dead cells (postbiotics) and probiotics. This need has also been emphasized by Boyte et al. [188], who specifically focused on the enumeration of beneficial microorganisms in various physiological conditions. Indeed, while probiotics, being living cell preparations, inevitably carry the burden of many, often unpredictable variables, postbiotics can and should be subject to appropriate quality control (QC) for the presence of each substance in their composition. This QC, enhanced by proteomic analysis [189], will take postbiotics to a completely different level of safety, reproducibility, and predictability of their effects (Figure 3).

Comparative analyses, such as those presented by Piqué et al. [190], have highlighted a growing number of reports indicating that inactivated probiotics can often be as active, or even more active, than their live counterparts. A recent and comprehensive systematic review by Kerksick et al. [191] also concluded that dead probiotic‐derived formulations exhibit higher safety and stability, delivering health benefits comparable to those observed with live probiotics in many in vivo studies.

Moreover, Kerksick et al. [191] identified only three studies that directly compared pro‐ and postbiotic preparations derived from the same strains, *Lpb. plantarum* TWK10, *Lbs. paracasei* PS23, and *Heyndrickxia* (formerly *Bacillus*) *coagulans* GBI‐30 6086. These studies revealed comparable, yet distinct, activities of the postbiotics relative to probiotics. However, no studies have examined the combination of pro‐ and postbiotic formulations to evaluate potential additive or synergistic effects. Furthermore, the authors noted the absence of dose‐response studies or investigations into how production methods and process conditions influence effectiveness.

## 7 Postbiotics: A Battle of Definitions, Challenges, and Future Perspectives

Postbiotics, which refer to the byproducts of probiotics or other microbial cultures, are gaining attention for their potential health benefits. However, several scientific and manufacturing challenges must be addressed for their successful commercialization and widespread adoption. Postbiotics can vary significantly in their composition depending on the microorganism, fermentation process, and environmental conditions. This lack of standardization makes it difficult to define what constitutes a “postbiotic” and hampers consistency in product formulations.

Identifying the specific bioactive components responsible for the health benefits of postbiotics is challenging. This requires advanced analytical techniques to isolate and characterize these molecules, which can be time‐consuming and costly.

Postbiotics face regulatory uncertainty. Different regulatory agencies (e.g., FDA, EFSA) may classify them differently, and a universal framework for their regulation has yet to be established. This regulatory uncertainty can slow down the development and approval of postbiotic‐based products. Manufacturers must demonstrate the health benefits of postbiotics to substantiate specific health claims, which requires extensive clinical testing. The lack of large‐scale postbiotic clinical trials means health claims are often limited or unsupported.

Many postbiotics are unstable, especially those derived from live microbial cultures. They may degrade over time, losing efficacy in the final product. Ensuring postbiotics maintain their activity and potency during manufacturing, storage, and transportation is challenging. Preserving postbiotics often requires specific conditions, such as refrigeration, which can add to the complexity of product development and increase costs.

Postbiotics are often produced through fermentation; however, scaling up fermentation processes from the laboratory to an industrial scale can introduce variability. Factors such as temperature, pH, and nutrient availability can affect the yield and composition of postbiotics.

Improving the efficiency of fermentation processes while maintaining the quality and consistency of the postbiotics is a significant challenge in manufacturing.

Although postbiotics are considered safer than live probiotics, there is still limited information on their long‐term safety, especially when consumed in large quantities. The safety profiles of various postbiotics require a more thorough study. Since postbiotics are often composed of complex mixtures of molecules, it can be challenging to assess their toxicity. Comprehensive toxicological evaluations are required to ensure that postbiotics are safe for human consumption, particularly in vulnerable populations.

Consumers are still relatively unaware of postbiotics compared to probiotics. Educating the public about the benefits and differences between probiotics and postbiotics is essential for market acceptance.

The scientific evidence supporting the health benefits of postbiotics is still limited. More clinical trials are needed to confirm their therapeutic potential and establish clear evidence of efficacy for various conditions, such as gut health, immune modulation, and metabolic diseases.

One of the primary benefits of postbiotics is their ability to interact with specific biological pathways but targeting them effectively to achieve therapeutic outcomes can be complex. Developing delivery systems that target the desired tissues or organs is an ongoing challenge.

While postbiotics hold great potential in various health applications, scientific and manufacturing challenges must be addressed before they can be widely adopted. Overcoming issues related to standardization, stability, scalability, safety, and clinical evidence will be key to unlocking their full potential in the market.

The evolving definition of postbiotics remains a topic of considerable debate. Concerns surrounding standardization and clarity have been highlighted by various researchers who have been pivotal in this discourse. A critical table from the literature [17] outlines these challenges and provides a comprehensive overview of definitions, production processes, and proposed applications. Future work must focus on harmonizing definitions to ensure consistent terminology and facilitate comparative research.

## 8 Conclusion

Postbiotics represent a promising area of research and application that bridges the gap between the benefits of live beneficial microorganisms (including probiotics) and the safety, stability, and efficacy of inactivated microbial formulations that include multiple bioactive metabolites and structural molecules. These inactivated microbial cells and/or their cellular components, along with their metabolites, have shown potential health benefits. However, to fully realize the potential of postbiotics, it is necessary to solve a number of scientific and manufacturing challenges that will ensure their successful commercialization and widespread implementation.

First, there is a pressing need for a standardized definition of postbiotics. Currently, the term “postbiotics” is used loosely, leading to confusion and inconsistency in research and application. Establishing clear criteria for what constitutes a postbiotic, including specific biomarkers for their identification and mechanisms of action, will help harmonize research efforts and ensure the reproducibility of findings. The development of such standards will also facilitate regulatory approval and consumer acceptance, paving the way for wider utilization of postbiotics in health‐promoting interventions.

Second, the implementation of appropriate technology for the deactivation of bacterial cultures is crucial. For this purpose, existing physical, chemical, and biological (e.g., enzymatic) methods are commonly utilized on a small scale, each of which has its own set of challenges. Mechanical methods, such as bead milling and ultrasonication, can generate heat and shear forces that may denature sensitive biomolecules. Chemical methods, involving detergents and solvents, can introduce contaminants and require careful handling to avoid adverse effects on the desired products. Biological methods, utilizing specific enzymes like lysozyme and proteases, offer a more targeted approach with minimal damage to intracellular biomolecules. Optimization of microbial fermentation processes, decontamination, and purification methods to preserve the integrity of beneficial metabolites and other active molecules is essential to maintaining the stability and industry‐acceptable shelf life of postbiotics for their efficient industrial production and widespread use as health‐promoting formulations. Advanced technologies and innovative preservation techniques should be explored to achieve these goals.

In conclusion, it should be noted that numerous types of biologically active microbial molecules, united under the general term “postbiotics,” hold great promise for improving human and animal health and developing targeted biotherapeutics. Further study of the most effective microbial producers of postbiotics, their mechanisms of action, and comparative analysis with probiotics, as well as the development of uniform definitions and an acceptable universal framework for their regulation, will be of decisive importance. The most important issue for industrial implementation is the need to develop microorganism inactivation technologies that will allow realizing the full potential of postbiotics as formulations aimed at modulating human and animal health.

## Funding

M.L.C. and X.L. were supported in part by the National Foreign Experts Individual Human Project, Grant number: H20240208; S.D.T. was supported in part by FAPESP (Grant 2023/05394‐9), Sao Paulo, SP, Brazil and FCT (UIDB/05937/2020 and UIDP/05937/2020), Fundação para a Ciência e a Tecnologia, Portugal; S.D.T. and M.L.C. were supported by Agência USP de Cooperação Acadêmica Nacional e Internacional (AUCANI, USP International Cooperation Office), Grant 1819/2023, University of Sao Paulo, Sao Paulo, SP, Brazil. V.L.F. was supported by CAPES 88887.941664/2024‐00.

## Ethics Statement

The current manuscript is a critical overview of the existing literature and does not contain experiments involving animals or humans.

## Conflicts of Interest

D.E.R. has equity in Nutrasorb, LLC. L.S.S. has equity in Stellar Biotics, LLC. J.R.T has equity in BLIS Technologies. The other authors declare no conflict of interest.

## Data Availability

The data that support the information based in this review paper is based on publiclly disponivel data.
